# The Prediction and Treatment of Bleeding Esophageal Varices in the Artificial Intelligence Era: A Review

**DOI:** 10.7759/cureus.55786

**Published:** 2024-03-08

**Authors:** María Isabel Murillo Pineda, Tania Siu Xiao, Edgar J Sanabria Herrera, Alberto Ayala Aguilar, David Arriaga Escamilla, Alejandra M Aleman Reyes, Andreina D Rojas Marron, Roberto R Fabila Lievano, Jessica J de Jesús Correa Gomez, Marily Martinez Ramirez

**Affiliations:** 1 Primary Care, Universidad Católica de Honduras, Tegucigalpa, HND; 2 Radiology, Thomas Jefferson University Hospital, Philadelphia, USA; 3 General Medicine, Universidad Nacional Autónoma de Honduras, Tegucigalpa, HND; 4 General Practice, Universidad del Noreste, Tampico, MEX; 5 Internal Medicine, Universidad Justo Sierra, Mexico City, MEX; 6 Internal Medicine, Universidad Católica de Honduras, Tegucigalpa, HND; 7 General Medicine, Universidad de Oriente, Barcelona, VEN; 8 General Medicine, Universidad Justo Sierra, Mexico City, MEX; 9 General Practice, Universidad Justo Sierra, Mexico City, MEX; 10 Internal Medicine, Universidad Nacional Autónoma de Mexico, Mexico City, MEX

**Keywords:** artificial intelligence, esophagogastroduodenoscopy, hemorrhagic, cirrhosis, esophageal varices

## Abstract

Esophageal varices (EVs), a significant complication of cirrhosis, present a considerable challenge in clinical practice due to their high risk of bleeding and associated morbidity and mortality. This manuscript explores the transformative role of artificial intelligence (AI) in the management of EV, particularly in enhancing diagnostic accuracy and predicting bleeding risks. It underscores the potential of AI in offering noninvasive, efficient alternatives to traditional diagnostic methods such as esophagogastroduodenoscopy (EGD). The complexity of EV management is highlighted, necessitating a multidisciplinary approach that includes pharmacological therapy, endoscopic interventions, and, in some cases, surgical options tailored to individual patient profiles. Additionally, the paper emphasizes the importance of integrating AI into medical education and practice, preparing healthcare professionals for the evolving landscape of medical technology. It projects a future where AI significantly influences the management of gastrointestinal bleeding, improving clinical decision-making, patient outcomes, and overall healthcare efficiency. The study advocates for a patient-centered approach in healthcare, balancing the incorporation of innovative technologies with ethical principles and the diverse needs of patients to optimize treatment efficacy and enhance healthcare accessibility.

## Introduction and background

Esophageal varices (EVs) are a severe complication of cirrhosis with an annual rate ranging from 5% to 8% [1,2]. In the United States, EV ranked as the seventh most common cause of gastrointestinal bleeding [3]. Cirrhosis manifests as a consequential outcome of chronic liver disease, distinguished by progressive fibrosis, scarring, and architectural distortion within the hepatic framework [4]. This condition can result in portal hypertension, which can lead to acute variceal bleeding, a life-threatening condition with a mortality rate of up to 20% [5-7]. For this reason, determining the risk of esophageal bleeding and effectively managing this condition are essential to decreasing hospitalization and mortality rates [1,8]. According to the World Gastroenterology Organisation, esophageal bleeding is associated with a 20% mortality rate at six weeks [2]. In addition, cirrhotic patients with bleeding events are associated with a hospitalization rate of 90% from the emergency room [9].

Esophageal bleeding results from the rupture of enlarged esophageal veins [1]. Therefore, the diameter of these veins can be helpful to predict the risk of bleeding [1,10]. In a recent study conducted in China in 2023, a noninvasive measurement device using artificial intelligence (AI) was utilized to measure the diameter and pressure of EV in seven patients [10]. This noninvasive device, a virtual ruler (VR), showed better performance in determining the diameter of EVs when compared with the esophageal varix manometer (EVM) [10].

Currently, esophagogastroduodenoscopy (EGD) is considered the gold standard method for assessing EV. However, this technique is costly and invasive, requiring the innovation of noninvasive methods [11]. In clinical practice, AI can be used to noninvasively diagnose EV and portal hypertension, which can aid in managing these conditions during their complete course [11-13]. Hence, using AI can be beneficial in predicting the risk of esophageal bleeding and, consequently, managing this complication more effectively. AI has been commonly used in clinical practice in many other ways nowadays. For instance, in a recent prospective study conducted in the United States in 2023, an ultrasound-based machine learning model using AI was used to detect metabolic dysfunction-associated steatotic liver disease (MASLD, formerly known as non-alcoholic fatty liver disease {NAFLD}) [14,15]. This machine learning device using ultrasound and AI reported high positive predictive value and specificity for detecting MASLD in high-risk patients [14].

This study aims to overview and highlight the role of AI in advancing noninvasive diagnostic methods and treatment strategies for bleeding EV due to the increase of AI use in the medical field and the need to summarize this information to provide better treatment to our patients.

## Review

Esophageal disorders: A clinical perspective

**Figure 1:**
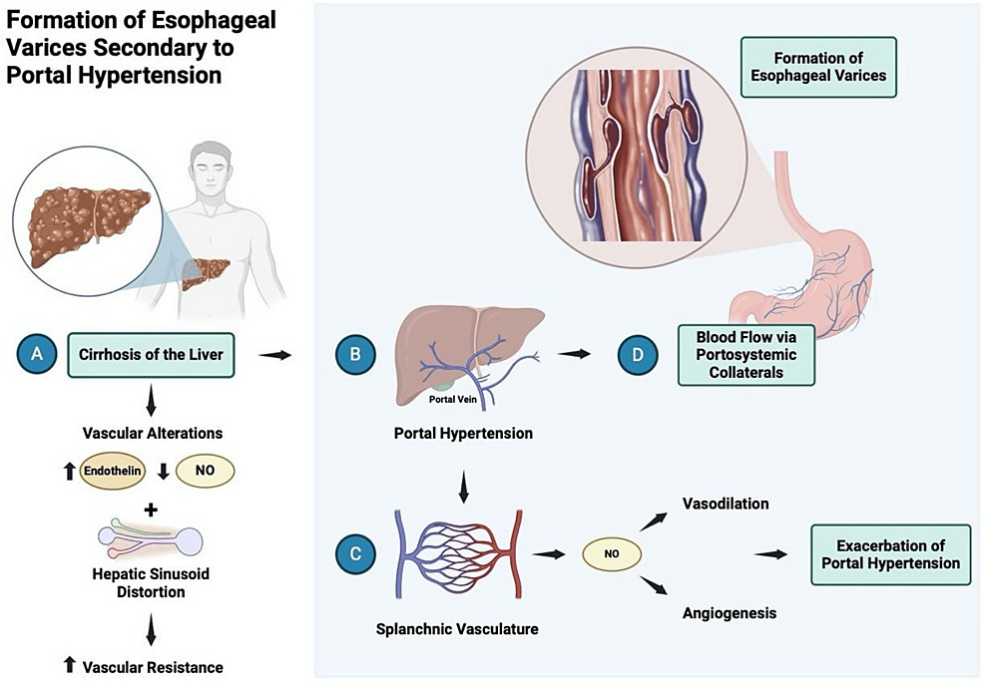
Formation of esophageal varices secondary to portal hypertension (A) Liver cirrhosis increases the vascular resistance of the liver through the mechanical distortion of hepatic sinusoids and vasoconstriction driven by decreased vasodilator availability (nitric oxide {NO}) and increased vasoconstrictor production (endothelin). (B) Increased vascular resistance in the liver and augmented flow from the splanchnic system result in elevated portal pressure. (C) Increased portal pressure signals to the splanchnic system to promote vasodilation and increase portal flow. Nitric oxide is recognized as a major player in mediating splanchnic vasodilation and angiogenesis. (D) Portal hypertension, in turn, feeds portosystemic collaterals and underlies the development of varices and ascites [19]. Figure made in BioRender; all credits to Maria Isabel Murillo Pineda

Upper gastrointestinal bleeding (UGIB) is a typical gastrointestinal emergency with a mortality rate of 5%-14% [16]. The esophageal etiology of UGIB includes erosive esophagitis, infectious esophagitis, pill-induced esophagitis, esophageal malignancy, Mallory-Weiss tear, black esophagus (ischemia), esophageal varices (EVs) [17] among others less common such as primary sclerosing cholangitis (PSC), or some syndromes [18]. EV is a portosystemic collateral, i.e., vascular channels that link the portal venous and the systemic venous circulation. They form due to portal hypertension, often in the submucosa of the lower esophagus (Figure 1) [2]. In clinical practice, EV can be classified as high risk or low risk (Table 1).

**Table 1:** Classification of esophageal varices

Classification	Characteristics
Low-risk varices	Small varices of <5 mm without red color signs [18]
High-risk varices	Medium-large varices of >5 mm. Small varices with red color signs (red wale markings, cherry-red spots, and hematocytic spots). Small varices in Child-Pugh class C cirrhotic patients [18]

In individuals with cirrhosis, esophageal varices (EVs) are a significant concern, accounting for about 10%-30% of all upper gastrointestinal bleeding (UGIB) cases in such patients [2] and nearly 70% of UGIB cases specifically in those with cirrhosis [20]. At the initial diagnosis, approximately 30% of cirrhotic patients have EVs, and this prevalence increases to nearly 90% over 10 years [2]. Despite medical advances, the mortality rate of EVs can be as high as 20% within the first six weeks after they occur [21]. Additionally, 9%-36% of EVs are classified as high risk for bleeding, and each year, 4%-30% of patients with small varices progress to large varices, increasing their bleeding risk [2]. While 40%-50% of variceal hemorrhages may resolve spontaneously, contemporary treatments successfully control bleeding in about 80% of cases [20].

EVs typically present with painless, effortless, recurrent hematemesis [22]; black, tarry, or bloody stools; lightheadedness; and loss of consciousness in severe cases [23]. The early diagnosis of EVs is crucial for clinicians to prevent the first bleeding episode, as studies confirm significant risk reduction with primary prevention. However, current clinical, biochemical, and radiological parameters often necessitate screening endoscopy due to their limited accuracy. Despite this, an assessment of systemic hemodynamics and serum markers holds promise for the future, with ongoing debate regarding the preferred primary prevention method: pharmacological or endoscopic. Both modalities are effective, especially in patients with cirrhosis and large varices, although endoscopy is preferred when beta-blockers are contraindicated. Acute variceal bleeding signals decompensation and high mortality risk in cirrhotic patients, requiring immediate volume resuscitation, vasoactive drugs, and antibiotics; approximately 10%-15% of cases do not respond to first-line therapies, with patient age and liver disease etiology influencing treatment decisions. This special issue offers a comprehensive overview and expert insights into current knowledge on the subject [24].

Prediction and early detection of bleeding esophageal varices (BEV)

Esophageal varices may constitute a life-threatening clinical emergency that poses a medical challenge; given its urgent nature, the timely identification of this complication and the use of several noninvasive or invasive tools are of paramount importance to allow the clinician to be able to predict its presence and detect any bleeding as soon as or even before it presents to avoid fatal outcomes. Even with the advent of new therapies and technologies, mortality remains around 12%-22% [1,25].

One of the most common risk factors for the development of bleeding esophageal varices is portal hypertensive gastropathy in patients with a history of liver disease or alcohol abuse. Variceal bleeding is strongly related to portal hypertension, hence the importance of measuring portal pressure in this group of patients [26]. In the assessment of portal pressure, the gold standard is the hepatic venous pressure gradient (HVPG, Table 2), a minimally invasive fluoroscopic technique that is measured by catheterizing the right jugular or femoral vein; clinically significant portal hypertension is greater than 10 mmHg. However, data suggests that HVPG may not always reflect the actual severity of portal hypertension [27,28]. Even though it can only be performed at advanced health centers, it has pivotal clinical utility in diagnosing portal hypertension and determining esophageal varices' endoscopic grade [29-31].

**Table 2:** HVPG interpretation HVPG: hepatic venous pressure gradient

Pressure	Interpretation
1-5 mmHg	Normal pressure
Greater than 5-10 mmHg	Portal hypertension without clinical signs
Greater than 10 mmHg	Clinically significant portal hypertension (CSPH)
Greater than 10-12 mmHg	Development of esophageal varices

Several methods have been proposed to identify variceal bleeding, stratify risk, or predict clinical outcomes, some of which are described below​.

Biochemical Tests

While no single biochemical test may predict esophageal varices or their worsening, some may estimate clinically significant portal hypertension. These biochemical tests are focused on liver fibrosis evaluation. Several markers have been linked to the development of esophageal varices, but data is inconclusive: laminin and glycoprotein. There is also a panel of biomarkers called FibroTest, which is the most validated indirect test for liver fibrosis that has a strong correlation with HVPG, especially in patients without liver cirrhosis; this panel includes five biomarkers: α2-macroglobulin, haptoglobin, apolipoprotein A1, bilirubin, and gamma-glutamyl transpeptidase (GGT).

One of the approaches is the aspartate aminotransferase (AST) to platelet ratio index (APRI) test. Although useful in identifying fibrosis, it is not a substitute for endoscopy in detecting esophageal varices [32]. Another approach is fibrosis-4 (FIB-4), based on four simple parameters: AST, alanine aminotransferase (ALT), platelet count, and age. Some authors point to its clinical relevance in avoiding variceal endoscopy, and others determined that it cannot be replaced by methods like this [32]. Another common laboratory test that helps is service albumin, as there is data associating low albumin levels with increased mortality in some patients with variceal bleeding and upper gastrointestinal bleeding as well.

We also have several scores that help in the assessment of each patient, which are summarized in Table 3.

**Table 3:** Scores for EV Evidence suggests AIMS65's superiority in predicting mortality over the Glasgow-Blatchford and Rockall scores [33,39] BUN, blood urea nitrogen; MELD, Model for End-Stage Liver Disease; GI, gastrointestinal

Score	Parameters	
AIMS65 score	Albumin, international normalized ratio (INR), altered mental status, blood pressure, and age	Predicts mortality, and evaluates the need for transfusion in patients with variceal bleeding; mortality risk is high when two or more are present [33,34]
Glasgow-Blatchford score	Blood pressure, hemoglobin, pulse, BUN, and melena or syncope	Identifies patients with esophageal varices (EVs) that need intervention, not so useful to predict outcomes [35]
Rockall score	Age, shock, comorbidity, the evidence of bleeding, and post-endoscopic diagnosis	Predicts mortality in patients with GI bleeding; some patients showed an increasing trend of rebleeding with increased Rockall scores [36]
MELD score	Bilirubin levels, creatinine, international normalized ratio, and the etiology of liver disease	Indicates short-term survival in patients with end-stage liver disease [37]
Child-Turcotte-Pugh score	Ascites, bilirubin, albumin, prothrombin time or international normalized ratio, and the presence or absence of encephalopathy	Assess severity of cirrhosis
Baveno VI criteria	Platelet count and liver stiffness measured by transient elastography	Good for ruling out high-risk varices [38]

Imaging and early diagnosis

Many efforts have been made to replace invasive methods in the early detection of portal hypertension, and imaging is essential to reach this goal.

Doppler Ultrasound

It provides real-time hepatic blood flow measurements. This method has been very useful, especially because it is noninvasive. Doppler ultrasound may help diagnose portal hypertension in non-advanced health centers that do not have access to HPVG measurement. It allows the measurement of the portal flow velocity, portal blood flow, and congestion index of the portal vein. Nevertheless, the downside of Doppler ultrasound is that this is an operator-dependent method and sometimes lacks the comparability of some parameters because of the use of different equipment [40]. With the advent of new technologies, pocket-size devices now allow us to have several options, from double-checking the side effects of medications to having a portable ultrasound. Point-of-care ultrasound (POCUS) is a useful tool that has broadened its clinical applications in the last few years because of new technological implementations in the medical field from examination to cardiology assessment [41]. Gastric ultrasound, in conjunction with a POCUS screening assessment as the Rapid Ultrasound for Shock and Hypotension (RUSH) protocol, has demonstrated its potential to detect early upper gastrointestinal bleeding. However, we still lack enough evidence to support this statement [42]. POCUS can be helpful in conjunction with ultrasound in the early detection of variceal bleeding because it is a simple, accessible, and affordable method. Nevertheless, more studies are needed to prove its role in this indication, and more staff need to be trained to provide the patient with the highest quality of attention [43,44].

Computed Tomography and Magnetic Resonance (MR)

Computed tomography is an excellent noninvasive alternative in the evaluation of portal hypertension. Its sensibility and specificity for detecting esophageal varices are 0.896 and 0.715, respectively; with new technologies, it is now possible to obtain three-dimensional (3D) images of the abdomen that provide similar accuracy as esophageal gastroduodenoscopy for the detection of esophageal varices [45]. Some imaging techniques allow the estimate of HPVG through azygos flow measurement [46]. 3D MR elastography (MRE) uses low-frequency waves in the abdomen to estimate hepatic fibrotic changes; this method is based on the principle that correlates liver and splenic stiffness with high portal pressure and HVPG. Evidence suggests that the more frequent use of MRE is recommended in esophageal varices' evaluations as a predictive tool [46].

AI to Predict and Detect Esophageal Varices

Machine learning algorithms, neural networks, and radiomics are fast-paced developing fields in medicine that, year after year, are showing promising results, especially in imaging interpretation. The creation of new software in detecting esophageal varices has shown comparable results with endoscopic findings; even though large-scale studies are still needed, an artificial intelligence called ENDOANGEL-GEV outperformed many endoscopist findings [47,48]. Several other software programs are still in development, but some neural network systems demonstrated comparable results with endoscopy predicting bleeding episodes [49]. Machine learning algorithms have taken the spotlight on predicting high-risk esophageal varices. This is the case for a novel score called EVendo, which considers several factors: the presence of clinical ascites, hemoglobin (g/dL), platelet count (count/1000), AST (U/L), and blood urea nitrogen (BUN) (mg/dL). EVendo score demonstrated its safety in clinical practice for predicting esophageal varices and varices needing treatment, with a higher number of spared endoscopies than with Baveno VI [50,51].

Esophagogastroduodenoscopy

The gold standard for diagnosing esophageal varices is the appropriate prevention method: diagnosing and treating bleeding episodes [52,53]. ​​​​​​

Treatment strategies for bleeding esophageal varices

Managing bleeding esophageal varices (BEV) is a complex problem, with many physiopathological conditions requiring an interdisciplinary approach. An acute episode of BEV has an early mortality rate of 20% during the first 24 hours, and complications will present in up to 40% of the patients; securing the airway, supportive interventions, and achieving hemodynamic stability in an intensive care unit are important for better outcomes [54-56].

Medical BEV (M-BEV) management should be individualized considering mortality scores, to provide a successful management, liberated or restricted transfusion strategy, the use of systemic and splanchnic vasoactive agents, discontinuation of non-selective beta-blockers (NSBB), restart of the latter, and the use of antibiotics. Tranexamic acid binds a lysine receptor in plasmin and inhibits fibrinolysis. A recent systematic review demonstrated how the early management of tranexamic acid has been shown to have positive outcomes in gastrointestinal bleeding and traumatic events; it reduces continued gastrointestinal bleeding and urgent endoscopic intervention but has not shown improvement in all causes of mortality [44,57,58].

Endoscopic variceal ligation (EVL) and endoscopic injection sclerotherapy have demonstrated in randomized controlled trials that their single overall outcome is better than other treatments, with decreased rebleeding, mortality, and variceal eradication; recent network meta-analysis and some multicenter retrospective studies have demonstrated EVL's superiority as a single treatment for active bleeding; the addition of other medical treatments could potentiate the overall results, such as the dual therapy of EVL and NSBB [56,59]. Transjugular intrahepatic portosystemic shunt (TIPS) has been shown to control and reduce some of the pathophysiology behind the BEV with high efficacy. Early TIPS has demonstrated that acute bleeding has been associated with decreased mortality and rebleeding [60,61].

Another promising therapeutic procedure has been the splenectomy plus selective pericardial devascularization, which offers a compensatory mechanism and physiologically compatible treatment [62]. Studies proved that treatment must be better oriented based on the Model for End-Stage Liver Disease (MELD) scores and Child-Pugh classification [54,61]. Early referral to an institution with an intensive care unit with a coordinated multidisciplinary approach and tailored treatment of the underlying disease, either cirrhosis or first BEV episode, by a team of anesthetists, hepatologists, gastroenterologists, hematologists, interventional radiologists, and other support teams is necessary to increase an overall outcome [54,61,63].

Medical education and AI integration: Preparing the healthcare workforce

AI plays a pivotal role in advancing patient care across various medical fields, such as radiology, dermatology, oncology, cardiology, orthopedics, and primary care. Beyond its current applications, AI holds the potential to improve health equity and quality by providing real-time health predictions and risk alerts and, consequently, reducing errors in diagnosis and treatment [64].

With the remarkable progress of AI in healthcare, particularly in the endoscopy field, AI has been used with colonoscopy to detect colorectal adenomas and esophagogastroduodenoscopy to minimize blind spots [47]. It is essential for medical education to continually incorporate advancements, ensuring healthcare professionals stay ahead of the latest, more accurate technologies. Therefore, it is imperative to integrate AI education into the medical curriculum. AI still has the potential to replace human decision-making with machine decision-making, undermining human authority. To preserve autonomy in a healthcare setting, physicians should learn to use AI tools, assess the accuracy of AI outcomes, and rework existing workflows. Since medical students have less experience than physicians, they tend to have more conservative views about AI [65].

AI in clinical settings requires health informaticists and physicians to undergo professional training for optimal application. Collaborating with engineers to create reliable AI applications necessitates a deep understanding of complex algorithms, data quality evaluation, probabilistic forecasting, and comparative model assessment. Physicians utilizing AI applications should be prepared for probable difficulties. Specialized training is needed to understand the errors made by AI algorithms and provide the appropriate plans of action to fix these applications. Therefore, the medical and health informatics education curriculum should include relevant themes such as data analytics, AI, and algorithm-based platforms, incorporating programs in computer science and health informatics [66].

Future directions

EGV represents a significant economic and population health issue [3]. The financial burden associated with hospitalization for variceal upper gastrointestinal bleeding (VUGIB) is substantial, reaching $23207 without complications and $6612 with complications. The average length of stay (LOS) is 15.2 and 3.8 days with and without complications/comorbidity, respectively. In contrast, hospitalization costs for non-variceal upper gastrointestinal bleeding (NVUGIB) stand at $5632 and $3402 with and without complications, respectively, with a mean LOS of 4.4 days with complications/comorbidity and 2.7 days without complications/comorbidity. There is substantial cost variation in the United States, even within the same city. Factors such as different treatment approaches, the rapid evolution of technologies, hospital differences, and patient characteristics contribute to this cost and LOS disparities [67].

For this reason, the future of gastrointestinal bleeding will include the integration of machine learning algorithms to enhance clinician risk assessment and decision-making. Utilizing data from electronic health records, machine learning algorithms can efficiently identify patients experiencing acute gastrointestinal bleeding. This enables an automated process to detect symptomatic patients, triggering risk prediction tools for consistent decision support to physicians. Neural network models can provide continuous risk predictions, aiding in triaging higher-risk patients to appropriate levels of care. Looking ahead, there is potential for a neural network-based analysis of endoscopic findings related to bleeding, contributing to best practices for hemostasis during endoscopic procedures. Overall, the integration of machine learning is anticipated to enhance care delivery at every level for patients with acute gastrointestinal bleeding [68].

AI can completely revolutionize healthcare practices by promoting more accurate diagnosis, individualized care, efficient resource allocation, improved accessibility to healthcare services, and streamlined clinical workflows. AI-enabled diagnostic algorithms demonstrated an average accuracy of 92%, surpassing traditional methods and paving the way for more precise and timely disease identification. Additionally, AI-optimized treatment plans led to a 20% increase in positive patient outcomes and a 25% reduction in hospital readmission rates, indicating improved treatment efficacy. Furthermore, AI-driven resource allocation strategies showcased a 15% decrease in hospital resource use and a 30% decrease in unnecessary tests, emphasizing enhanced efficacy and cost-effectiveness. Additionally, introducing AI-powered telehealth platforms led to a 40% rise in remote consultations, improving underserved communities' access to healthcare services [69].

Variceal hemorrhage remains a significant medical challenge that requires ongoing research and the development of new treatment approaches. Optimal advancements in variceal bleeding management necessitate the implementation of large-scale clinical trials, prospective cohort studies, and exploring innovative therapeutic approaches and technological applications [70]. These endeavors are imperative not only to enhance patient outcomes but also to establish evidence-based guidelines [71]. The effective management of bleeding esophageal varices requires a holistic approach considering ethical principles, regulatory standards, and patient-centered care [72]. By balancing these factors, medical interventions are guaranteed to be clinically successful and aligned with the values and preferences of the individuals receiving care. This reflective approach contributes to a comprehensive and compassionate model of healthcare delivery [73].

## Conclusions

This manuscript highlights the critical nature of EV, a major complication of cirrhosis, emphasizing the role of AI in advancing diagnostic and management strategies. AI offers a promising avenue for noninvasive, accurate prediction and the early detection of EV, potentially surpassing traditional methods such as esophagogastroduodenoscopy (EGD). However, managing EV remains complex, requiring a combination of pharmacological, endoscopic, and surgical approaches tailored to individual patient needs. The integration of AI in medical practice underscores the need for ongoing education and adaptation among healthcare professionals. Future directions in gastrointestinal bleeding management are poised to leverage AI for enhanced decision-making, aiming to improve patient outcomes and healthcare efficiency. This evolution in healthcare technology, while promising, must be approached with a comprehensive understanding of its implications in clinical practice.
